# Clinical Aspect of Some Disorders of the Gastro Intestinal Tract of the Horse*Read before the Keystone Vet. Med. Association of Philadelphia, Aug. 13th, ’85.

**Published:** 1885-10

**Authors:** Thomas B. Rogers


### ART. XXIV.—CLINICAL ASPECT OF SOME DISORDERS OF THE GASTRO INTESTINAL TRACT OF THE HORSE.*

BY THOMAS B. ROGERS, V.S.

I wish to call your attention this evening to some diseases of the digestive tract of the horse—diseases acute in character and often fatal in termination. Endowed with a lengthy, vascular and highly sensitive alimentary canal, compelled to travel at speed, when he should be digesting at leisure a meal of well

---

* Read before the Keystone Vet. Med. Association of Philadelphia, Aug. 13th, '85.

masticated food, watered when the fluid should be withheld, or deprived of water when it would aid digestion, there is small wonder that "colic" in the horse occupies an analogous position to the diseases of the respiratory apparatus in man as the great factor in the bills of mortality. I shall not inflict upon you a detailed treatise on these conditions, and shall make but little reference to the pathological changes involved, feeling that I am not at all competent to present them at first-hand, and knowing that a réchauffé of the observations of others would have small interest for you.

*Causation.*—The causes of acute digestive troubles in the horse are varied: improper food, sweet potatoes, new wheat, new corn (some of the most violent cases I have seen were caused by feeding new wheat to horses working in threshing machines), improperly cured food, musty hay, mildewed grain, hay burnt or heated. Food given at improper times or in improper quantities. Speeding a horse immediately after a meal, or the fear and excitement incident to the abuse of a brutal driver may stop digestion. Irregularities of the teeth, bolting the food, cribbing, diseases of the parotids interfering with the secretion of saliva, paresis of the muscular coat of the bowel, the presence of a calculus or stenosis of the canal are all in casual relation to acute digestive troubles.

*Symptoms.*—Pain is common to all of them, varying in intensity from slight uneasiness to violent torment—the intensity of the pain is usually in inverse ratio to the gravity of the disorder. The more persistent the pain, whatever be its grade the graver the outlook. The conditions grouped as "colic" are mostly marked by intermissions, or at least remissions of the pain, the pain of colic induced by hepatic derangements where we often have localized peritonitis is the most persistent. The urine is usually retained (in the colicky symptoms preceeding rheumatic influenza there is often suppression), either from spasm of the sphincter vesical or from the preliminary straddling, causing pain. The rectal contents may be drier than normal, may be balled, fluid, or semi-fluid in consistence—they are usually ill smelling—the mucous membrane dry, its secretion suspended, or more than the natural quantity of rectal mucus may be found. The belly may be distended from evolved gases—this is not very observable if confined to the stomach or small intestines, but is readily noticed when the colon or cæcum are distended. Sweating is often seen if it is warm, and breaks out on the parts where it is first observed; on exercise it is of little moment; if cold and localized it usually indicates mechanical trouble beneath its seat; if cold and diffuse it is too often the forerunner of death. Nothing is more common than to hear a practitioner assert that a patient had colic, that the colic was followed by enteritis and death; the autopsy showing acute intestinal imflammation, all this taking place in about twelve hours. It is entirely fair here to assume one of two things—either a mistake in the original diagnosis, or an incorrect reading of the pathological conditions observed on post mortem. The symptoms of enteritis are elevated temperature, hot dry skin, pain not well marked, constipation often followed by diarrhœa, mucous or croupous discharges per anum, scanty and high-colored urine; the horse is dull, the pulse quick, soft and compressible; he is uneasy but not violent; he gets up and down carefully, and often stands in one position for many hours, and is sicker than he looks. The colicky horse exhibits a violence unseen in enteritis. His full, strong pulse shows his heart's action unimpaired; his temperature is not elevated; *rectal examination gives the colicky horse relief; in enteritis it gives pain.* The pain of colic is rapidly relieved by opiates, and with relief comes the desire for food; the relief afforded by opiates in enteritis is but partial, and is accompanied by no desire to eat. But, you say, what about the post mortem appearances? Just this! Part of the discoloration is due to post mortem staining and hypostatic congestion, part of it to ante mortem congestion and extravasation from the compressed vessels; acute inflammatory troubles don't run their course from start to finish in a few hours. Another point—the first symptoms of colic are rarely seen in the early morning hours; it is rare that a horse eats his breakfast with a relish, and is attacked with colic before noon. I do not recollect ever being called to a case commencing before noon. This is my experience, it may differ from yours, I give it for what it is worth. In peritonitis there is pain on pressure, and on rectal examination a quick wiry pulse, elevated temperature, *thoracic respiration, the flank muscles outlined and fixed,* and the fæcies hippocratica is as marked in the horse as in man; the eyes look sunken as if from absorption of their fatty cushion, and as the disease goes on there is œdema of the sheath, mammae and belly often extending between the front legs. I think its degree indicates the gravity of the disorder. You will find in your text books that great stress is laid on the acuteness of the pain, especially on pressure. I recently had the opportunity of watching a case of diffuse peritonitis (following the operation for strangulated scrotal hernia) from its commencement to its termination. At no time was the pain marked, at no time was it much augmented by pressure; the temperature stood from 103°–104° F. for several days; the horse ate fairly well until the last two days of life; he moved about freely and did not arch the back; the excretæ (rectal) were natural for some days; then, thickly coated with mucus (extension of the inflammation to the mucosa through contiguity?) the skin was pleasant to the touch; the temperature of the extremeties irregular; the pulse intermitted; the œdema was very great and progressive. Fortunately acute idiopathic peritonitis is very rare in the horse.

The colicky pain accompanying liver trouble is somewhat acute. Almost persistent the horse lies on his right side or back quietly, and takes good care of himself; the fæces are balled and clay colored, coated with mucus or semi-fluid, and tarry; the pulse is *slow and full;* the mucous membranes icteric or pale; the appetite not altogether lost; the urine high colored and scanty. In these cases the pain evinced on percussion over the lumbar insertion of the diaphraghm is a valuable symptom; it is not present in ordinary colic, it has never been absent in the colic due to liver derangement in my experience. It must be remembered that rupture of the stomach may complicate colic, it may be present when the veterinarian is called in, it may occur during treatment, and it is important to the practitioner's reputation that its presence should be detected. I was taught that vomiting rarely occurred in the horse save from ruptured stomach or aconite poisoning. Vomiting is certainly rare in the horse. I think I have seen four cases. They all got well. One sat on his hind legs like a dog, and vomited very freely, and I do not see how a horse can vomit when the contents of the stomach have escaped into the peritoneal cavity.

Substitute retching for vomiting, evident nausea, the ejection at intervals, of saliva and mucus, add to this the fæcies grippe, and a quick, weak thready pulse and you have a train of symptoms absolutely diagnostic of ruptured stomach. I do not say the gentlemen who have made a diagnosis of ruptured stomach from the occurrence of vomiting have mistaken post for ante mortem rupture, but I do insist that the rupture had not taken place when the vomiting occurred. I saw five cases last summer, diagnosed the condition, and verified it by an autopsy, and it is very instructive to observe how rapidly false membranes form around the seat of the rupture. Death takes place in twelve to twenty-four hours—its cause is probably shock.

*Intussusception* may be primary or occur in the course of an attack of acute indigestion, the pain is constant, there is straining, fæces back of the obstruction are passed, opiates fail of full relief, there is great anxiety and uneasiness and after a while a symptom of great diagnostic value will usually be observed, the presence in the recturn of bloody mucus due to extravasation from the compressed vessels. The prognosis in these cases is grave, though they are not absolutely fatal. I saw a case when in college where a foot of gut sloughed off and was discharged per anum, the horse making a good recovery. Again the parts may assume their natural position without our interference, through the altered pressure relations caused by distention of the bowel with gas, or conversely, its puncture. I relieved a case where I am as well satisfied that I had intussusception, as a man can be of anything he cannot see, by the use of the trocar.

In twisting of the gut the pain is intense, peritonitis follows, the parts swell, compression gives nerve pain, cold sweats occur over the part, the horse often walks a circle presistently, and if laying down assumes the dorsal decubitus, the pulse grows weak and thready, the extremities cold, the pain increases, is agonizing, unbearable, it stops suddenly, the breath is cold or fœted, the surface temperature runs down, and mortification has set in. The horse may now eat a little, and I saw a case where with a delirious neigh an animal fell back dead with a wisp of hay in his mouth. Not infrequently an attack of rheumatic influenza is ushered in by colicky pains and these cases are at first somewhat difficult of diagnosis. In engorgement the pain is not very marked. If the stomach is the seat there may be vomiting or spasmodic movements of the muscles of the neck showing nausea, the horse is full and sleepy and often rests his head on the manger. In engorgement of the small intestine relief is apparently afforded by backing up against a wall or fence, the intestinal murmur is lost, the temperature a little elevated, the muscles of the face somewhat drawn. I recently saw three cases closely simulating the "stomach staggers" of the old writers due to feeding freshly cut forage containing large quantities of "wild carrot," the pupils were dilated, the skin hot and dry, and the mental faculties evidently affected.

The presence of calculus gives rise to similar symptoms of mechanical obstruction as in engorgement or intussusception. Rarely its presence may be detected by manual examination, more rarely still it is voided per rectum.

*Treatment.*—No fixed rules can be laid down for the treatment of these cases, the routine practitioner must necessarily lose a good many of them. Factions have rallied alike around the sedative and purgative lines of treatment; the treatment must be adapted to the case. It is no use to stand over a horse and philosophise about the use of opium, while he is beating out his brains in the acuteness of his agony. He would die before a purge acted. On the other hand, if the pain is not acute or inflammatory, even if it persists for hours, the judicious practitioner will watch it and await the action of his purgative with confidence. I often give a full dose of opium or morphia and follow it by a purgative.

In impaction I rely on linseed oil and turpentine with bella donna Eserine has failed me entirely and several of my friends have had a similar experience with it, though Professor Zuill tells us that the addition of one or two drachms of powdered calabar bean to an aloes ball greatly quickens and facilitates its action, the drug however must be good. To get rid of flatus, charcoal, soda carbonate or bicarbonate, soda sulphite all commend themselves. I have seen great benefit from the administration of a ball of bicarbonate of ammonia, but care must be taken that only the hard translucent portion of the drug is used or it will fail you. If the accumulation of gas is in the stomach or small intestines these measures will be of service, but if in the colon or cæcum they are almost useless; here frequent enemas of soap and water, with a little, very little, turpentine, will be found very beneficial and the best instrument to give them with is three feet of inch rubber hose, having a funnel in one end and the other end in the rectum. An injection can thus be given "safely, quickly, pleasantly." This instrument should supplant the syringe entirely. The bladder is often full in the mare, and it may be emptied by bearing in mind that in the lower animals irritation of the external genitals causes contraction of the bladder, by inserting two fingers into the urethra and separating them repeatedly.

In the horse gentle pressure from the funders of the bladder toward its neck will usually cause urination, if it does not, the injection of four ounces of bland oil with an ounce of chloroform into the rectum will cause sufficient relaxation of the sphincter to allow the organ to be emptied by proper and repeated pressure.

In the July number of The Journal of Comparative Medicine and Surgery, Mr. Chas. J. Byrne says: "I have used the trocar in thirty-one instances with nine successful results." I have used it more frequently and had no deaths. When should the trocar be used? If the swelling does not respond rapidly to treatment and the intestinal murmur is absent and there is interference with respiration it should be used at once I have punctured a horse four times in a few hours and saved him, but it is no use waiting until an animal is in the shadow of death, and then using an instrument as big as a kitchen poker. The point of election is the seat of the greatest distention wherever that may be. I have punctured on the floor of the belly several times though I do not like it, as abscess follows more frequently on this than on the flank operation. Medicine should not be injected through the canula. The operation should not be undertaken rashly, as the judgement showing its necessity is the growth of experience. The young practitioner cannot get this from his books any more than he can learn to keep half a dozen balls in the air at a time by reading how it is done. When in doubt it is better to be too early than too late. I differ with Mr. Byrne on the question of exercise. I like my "colic" cases kept on the move unless there is weakness of the heart. I can give no better reason for this than the statement that in my experience a colicky horse lying down is usually a horse getting worse. The patient must not be left too soon, the symptoms may abate and return, and care should be had about his diet and work for a few days.

